# Open questions: why should we care about ER-phagy and ER remodelling?

**DOI:** 10.1186/s12915-018-0603-7

**Published:** 2018-11-01

**Authors:** Ivan Dikic

**Affiliations:** Institute for Biochemistry II and Frankfurt Cancer Institute, Goethe University, Theodor Stern Kai 7, 60590 Frankfurt, Germany

## Abstract

The endoplasmic reticulum (ER) is one of the most complex organelles in the eukaryotic cell. Recent findings suggest that a process called ER-phagy plays a major role in maintaining the ER’s shape and function.

## Shaping the ER

The endoplasmic reticulum (ER) comprises a continuous system of membrane sheets, tubules, and matrices, as well as specialized contact sites to other organelles. These substructures harbour processes that are as varied as they are important. The ER lumen functions as the main cellular Ca^2+^ store. The rough ER is studded with ribosomes that co-translationally insert nascent polypeptide chains, for both ER resident proteins and secretory proteins, into the ER lumen, where chaperones assist the correct folding process. The smooth ER is the site where lipids and steroid hormones are synthesized; it serves as an initiation site for autophagic membranes and as a hub for detoxifying enzyme activity. Mitochondria-associated ER membranes (MAMs), an ER contact site, exchange molecules and ions between ER and mitochondria and control the functional status of the reciprocal organelles.

Shape and function of the ER can be adjusted to the specific needs of different cell types. A prime example is from muscle cells, which contain sarcoplasmic reticulum optimized for serving as the major source of calcium release during muscle contraction. In contrast, secretory cells, like the acinar cells of the adult exocrine pancreas, have expanded rough ER to facilitate high levels of protein biosynthesis.

Yet, the ER also needs to react to rapid changes in protein and lipid demand, pharmacological insults, or pathogen attacks. An essential part of these dynamic changes is the restoration of the original pre-stress state, which requires dismantling of excess ER, removal of damaged parts, and degradation of ER components produced during the stress phase. In fact, a failure in proper ER shaping/remodelling and ER homeostasis is harmful to cells and appears to be a common mechanism underlying several human diseases, including infectious and neurodegenerative diseases and cancer.

## An autophagy pathway for maintaining the ER

**Fig. 1.**
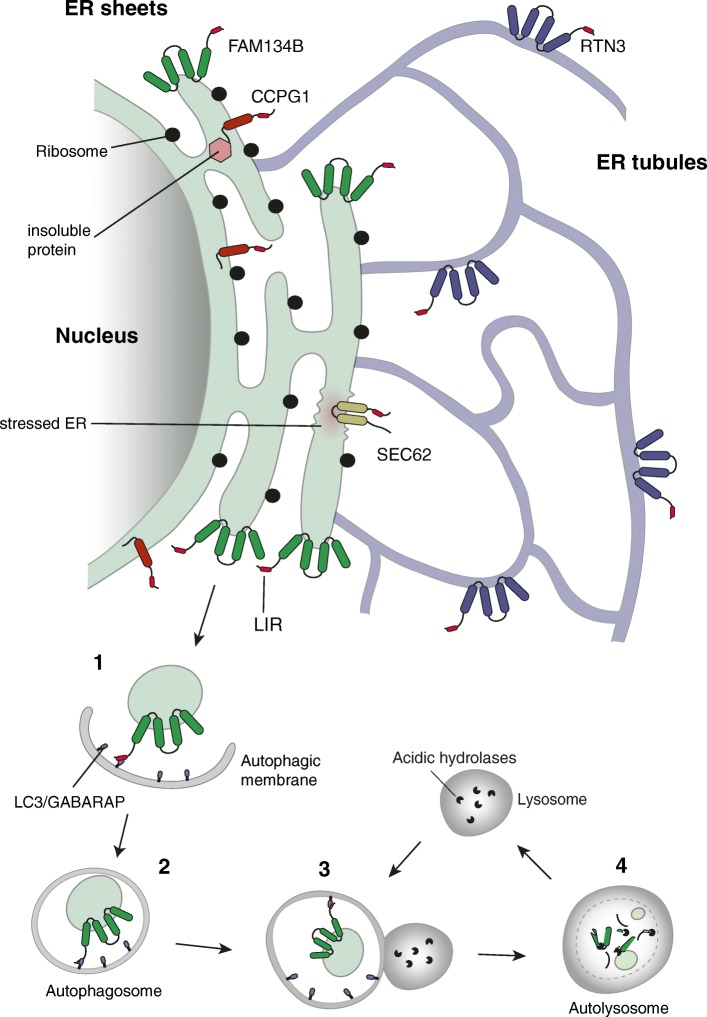
ER-phagy is mediated by ER-phagy receptors localized to distinct subdomains of the ER. FAM134B is restricted to the curved edges of ER sheets. RTN3 is found exclusively on ER tubules. Sec62 is localized to ER sheets following ER-stress. CCPG1 is found in areas of the ER with high content of insoluble proteins. The currently known receptors do not interact or functionally cooperate with each other. All of them contain LIR domains that are able to bind to LC3/GABARAP-decorated autophagic membranes. The process of ER-phagy can be summarized in four steps: *1*) cargo sequestration via interaction between LIR and LC3/GABARAP; *2*) closure of the autophagic membrane (aka isolation membrane) around the cargo; *3*) fusion of the resulting autophagosome with the lysosome; *4*) degradation of the enclosed ER fragments by lysosomal hydrolases and acidic pH

Within the past two decades, major proteins and cellular processes that shape the ER and maintain its characteristic substructures have been identified. The latest addition to this repertoire has been ER-phagy, whose role is to selectively remove unwanted portions of the ER. Like other selective autophagy pathways, such as mitophagy or ribophagy, ER-phagy not only is able to discriminate between still functional structures and damaged or excess material, but also deals with very heterogenous and often bulky material. The autophagic machinery, which enables this, orchestrates the sequestration of cellular content via a double-membrane-bound vesicle, the autophagosome, and mediates its fusion with the lysosome for cargo degradation (Fig. 1).

Initial studies indicated that ER-phagy plays a role in the basal turnover of the ER, in its re-shaping after expansion upon stress, and in the lysosomal degradation of protein aggregates within the ER lumen. Thus, ER-phagy occurs constantly at a low level under basal conditions to maintain ER homeostasis. Upon certain stimuli, such as ER stress, nutrient deprivation, accumulation of misfolded proteins, or pathogen attack, ER-phagy can be significantly increased [1].

In order to achieve selectivity, the autophagic machinery employs (i) specific labels, e.g., ubiquitin, which are attached to cargo, and (ii) selective autophagy receptors that recognize the label and link the cargo to the autophagic membrane. Cargo selection can also be mediated by autophagy receptors that are themselves part of the targeted organelle and become activated and/or surface-exposed when autophagic degradation is induced. This latter mechanism of cargo selection seems to be the predominant one in ER-phagy. The key feature of both types of autophagy receptors is the presence of one or more LC3/GABARAP-interacting regions (LIRs; AIM in yeast—Atg8-interacting motif), a specific sequence of four amino acids, that mediate the coupling to the autophagic membrane via binding to the ubiquitin-like LC3/GABARAP exposed on it. The understanding of ER-phagy, in terms of its physiological significance and mechanistic processes, is still at its infancy and a number of intriguing questions remain to be elucidated.

## What are the receptors and their cargos?

The knowledge of selective ER-phagy receptors is crucially important to uncover the physiological significance of ER-phagy. Up to now, four ER-resident proteins have been identified that serve as selective autophagy receptors in mammals: FAM134B, RTN3, SEC62, and CCPG1. FAM134B and RTN3 possess reticulon homology domains (RHD) that are inserted into the membrane such that the membrane is bent. Accordingly, FAM134B and RTN3 are localized at the curved edges of ER sheets and ER tubules, respectively, and their role in ER-phagy is restricted to the subdomains in which they reside [2, 3]. It is thought that FAM134B and RTN3 might actively participate in ER fragmentation with the help of their RHDs and then—via their LIRs—link the ER fragments to autophagic membranes. Consistently, down-regulation of FAM134B, or mutations in its LIR, cause an expansion of the ER, while FAM134B overexpression results in ER fragmentation and lysosomal degradation of ER sheets, including their resident proteins such as CLIMP63. Likewise, ER tubules are degraded in a RTN3-dependent manner. FAM134B and RTN3 play an essential role in degrading the ER in response to starvation or stress conditions to the ER. Moreover, misfolded NPC1, a structurally complex 13 transmembrane domain glycoprotein synthesized in the ER, was recently identified as an endogenous substrate for FAM134B-dependent ER-phagy [4]. Though it is currently unknown how FAM134B senses (or even directly recognizes) misfolded NPC1, this finding suggests that ER-phagy may also target specific misfolded proteins.

SEC62 was first known as part of the SEC61/SEC62/SEC63 translocation machinery which is located in the rough ER membrane and plays a central role in translocation of nascent and newly synthesized precursor polypeptides into the ER. Only recently it was discovered that SEC62—independently of its function in the translocation complex—acts as autophagy receptor in ER-phagy to support recovery of cells from ER stress [5]. Precisely, after ER stress resolution, its task is to remove excess ER components resulting from the activation of the unfolded protein response (UPR). Intriguingly, SEC62-mediated ER-phagy targets only those ER subdomains that contain molecular chaperones and folding enzymes that have been enriched during UPR, while other ER activities, for example ER-associated degradation (ERAD), are left untouched.

Besides FAM134B, RTN3, and SEC62, another ER-phagy receptor induced by ER stress is cell-cycle progression gene 1 (CCPG1) [6]. This transmembrane protein is localized to the perinuclear ER as well as in the ER periphery. In contrast to SEC62, whose major function seems to be the re-setting of the ER to pre-stress condition, CCPG1 locally restricts ER stress and maintains ER proteostasis by removing portions of ER carrying insoluble proteins. Pancreatic acinar cells of mice lacking CCPG1 accumulate ER that is loaded with insoluble zymogen protein, and ultimately undergo cell death, which potentially triggers pancreatic inflammation. The upregulation of unselective bulk autophagy has long been known to be a consequence of ER stress. However, SEC62 and CCPG1 represent the first direct links between ER stress and the targeted trimming of ER.

Given the structural complexity and many functions of the ER, it is very likely that the future will witness the discovery of many more selective ER-phagy receptors, as well as associated functions. For example, ER-phagy has been proposed to limit the replication of viruses (such as Ebola, Dengue, or Zika virus). In fact, viruses have evolved strategies to destroy FAM134B, subverting ER-phagy and escaping elimination [7]. However, whether FAM134B indeed functions as the sole ER-phagy receptor acting in anti-viral ER-phagy remains to be investigated. ER-phagy also serves as a microbial defence mechanism. There is strong evidence that ER stress-mediated ER-phagy is a cell-autonomous response to intracellular pathogens that utilize the ER for replication [8]. The ER-resident stimulator of interferon genes (STING) senses living microbes, activates ER stress, and subsequently ER-phagy, to eliminate affected areas of the ER. However, the involved ER-phagy receptor remains to be elucidated. Moreover, other ER structures such as the contact sites to other organelles or the perinuclear ER might also be selectively degraded via ER-phagy.

It is likely that ER-phagy variants exist that utilize specialized receptors optimized for the physiological demand of certain cell types such as neurons, kidney, or immune cells. This notion is corroborated by the fact that loss-of-function mutations in FAM134B primarily affect sensory neurons whereas other cell types, though rich in ER sheets, are not significantly impaired. This suggests that in these cell types other autophagy receptors are in charge.

## Are ER-phagy receptors team players or lone warriors?

Selective autophagy receptors operating in other autophagy pathways such as mitophagy or xenophagy often cooperate with each other for efficient cargo removal. In contrast, previous findings indicate that each ER subdomain, physiological condition, or ER activity utilizes one specialized ER-phagy receptor, which drives degradation of the structure it resides in. Indeed, no physical or functional interactions between the currently known ER-phagy receptors have been detected. However, FAM134B and RTN3 do not possess intraluminal domains that could sense a specific physiological condition or cargo requiring the induction of ER-phagy. Therefore, it is likely that accessory proteins team up with FAM134B and RTN3 to facilitate efficient ER-phagy. The nature of these accessory proteins may differ in various cell types and might be responsible for sequestering certain cargo into autophagosomes.

Another challenge in ER-phagy is to isolate only portions of ER from the highly interconnected ER membrane network to allow their subsequent degradation. Though it had been speculated that FAM134B and RTN3 may be able to fragment membranes via their RHDs, an ability both proteins demonstrate, it was recently shown that members of a family of GTPases called atlastins can take over this task downstream of FAM134B [9]. Atlastins are known to mediate tubule fusion and play an important role in shaping the ER. It remains to be tested whether FAM134B and RTN3 strictly depend on atlastins or whether, in certain conditions, they can autonomously fragment ER. In any case, it is very likely that SEC62 and CCPG1 need to cooperate with atlastins or similar factors, too.

## Future perspectives on ER-phagy

Studying the mechanisms of ER-phagy will not only shed light on a fundamental cellular process but also has medical implications as a number of human diseases have been linked with disturbed ER-phagy. As already mentioned above, ER-phagy plays a prominent role in innate defense against viral and bacterial infections [7, 10], whereas CCPG1’s function in pancreatic acinar cells implies an involvement in pancreatic disorders [6]. In addition, FAM134B and SEC62 have been linked to several types of cancer, suggesting that ER homeostasis and ER stress tolerance play a role in controlling tumorigenesis. Moreover, mutations in FAM134B and Atlastin-3 have been shown to affect the survival of sensory and autonomic neurons leading to the hereditary sensory neuropathy HSAN-II [2]. In each of these examples, a comprehensive understanding of the triggers and their consequences, on both the molecular and cellular level, will be an important task for the future.

Previous studies suggest that ER-phagy is a highly dynamic process that engages different parts of the ER via different molecular mechanisms, each involving one of the four currently known ER-phagy receptors (with more to be identified in the near future). However, it is currently unclear how exactly these receptors are activated, i.e. what causes them to bind to LC3 in a certain moment, and how they recognize intraluminal cargo. Different cell types and different stimuli may profit from a combinatorial receptor engagement: a main receptor (such as FAM134B or RTN3 that can actively bend and remodel ER membranes) might team up with different co-receptors that provide the link to a subset of signals. This appealing concept would provide a simple way of multiplying the same machinery for different types of cargoes. The identification and characterization of more ER-phagy receptors as well their interplay with other ER proteins and ER-phagy receptors will shed light on this aspect and also unravel the extent to which ER-phagy contributes to the shape and function of the ER. In fact, ER-phagy possesses the potential to remodel or rebalance the entire ER network and, given the physical and functional connection to virtually every organelle inside the cell via contact sites such as MAMs, ER-phagy might also impact the function of other organelles as well.
